# Towards a global monitoring system for implementing the Rio Political Declaration on Social Determinants of Health: developing a core set of indicators for government action on the social determinants of health to improve health equity

**DOI:** 10.1186/s12939-018-0836-7

**Published:** 2018-09-05

**Authors:** Working Group for Monitoring Action on the Social Determinants of Health, Aluisio Barros, Abdesslam Boutayeb, Christine Brown, Hazel D. Dean, Erica Di Ruggiero, Rita M. Ferrelli, Patricia Frenz, John Glover, Mana Herel, James Humuza, Doris Kirigia, Patricia O’Campo, Frank Pega, Srinath Reddy, Agata Stankiewicz, Tone Torgesen, Nicole B. Valentine, Eugenio Villar

**Affiliations:** University of Toronto and Chair of the Working Group for Monitoring Action on the Social Determinants of Health, Toronto, Canada

**Keywords:** Social determinants of health, Quality/process indicators, health care, Health status disparities, Equity, Rio declaration action areas, Policymaking

## Abstract

**Background:**

In the 2011 Rio Political Declaration on Social Determinants of Health, World Health Organization (WHO) Member States pledged action in five areas crucial for addressing health inequities. Their pledges referred to better governance for health and development, greater participation in policymaking and implementation, further reorientation of the health sector towards reducing health inequities, strengthening of global governance and collaboration, and monitoring progress and increasing accountability. WHO is developing a global system for monitoring governments’ and international organizations’ actions on the social determinants of health (SDH) to increase transparency and accountability, and to guide implementation, in alignment with broader health and development policy frameworks, including the universal health coverage and Sustainable Development Goals (SDG) agendas. We describe the selection of indicators proposed to be part of the initial WHO global system for monitoring action on the SDH.

**Methods:**

An interdisciplinary working group was established by WHO, the Public Health Agency of Canada, and the Canadian Institutes of Health Research—Institute of Population and Public Health. We describe the processes and criteria used for selecting SDH action indicators that were of high quality and the described the challenges encountered in creating a set of metrics for capturing government action on addressing the Rio Political Declaration’s five Action Areas.

**Results:**

We developed 19 measurement concepts, identified and screened 20 indicator databases and systems, including the 223 SDG indicators, and applied strong criteria for selecting indicators for the core indicator set. We identified 36 suitable existing indicators, which were often SDG indicators.

**Conclusions:**

Lessons learnt included the importance of ensuring diversity of the working group and always focusing on health equity; challenges included the relative dearth of data and indicators on some key interventions and capturing the context and level of implementation of indicator interventions.

## Background

In 2008, the World Health Organization’s (WHO) global Commission on Social Determinants of Health (CSDH) called for action on the social determinants of health (SDH), the conditions in which persons are born, grow, work, live, and age, to “close the gap in a generation” [1] (p1). Widespread recognition now exists that taking action on SDH is crucial for reducing and reversing the growing health inequities (i.e., unfair, remediable inequalities in health) [2] that exist within and between countries.

In 2011, a total of 125 countries developed and signed the *Rio Political Declaration* on *Social Determinants of Health* (hereafter Rio Political Declaration) [3]. The declaration recommended interventions from governments and international organizations in five Action Areas (Table 1). Building directly on and aligning closely with CSDH’s final recommendations [1], the declaration encompasses 50 pledges for implementing a minimum set of actions that address SDH for improving health equity across diverse sectors. The Rio Political Declaration’s vision of intersectoral and multisectoral action for health was endorsed by the 194 WHO Member States at the 65th World Health Assembly in 2012 (Resolution WHO65.8), and then echoed by 193 Member States of the United Nations (UN) in the 2015–2030 Sustainable Development Goals (SDGs) [4].

**Table 1:** Five Action Areas of the 2012 Rio Political Declaration on Social Determinants of Health

	Action Area
1	Adopt better governance for health and development
2	Promote participation in policymaking and implementation
3	Further reorient the health sector towards promoting health and reducing health inequities
4	Strengthen global governance and collaboration
5	Monitor progress and increase accountability

Source: WHO, 2011

The interventions on the SDH, recommended in the pledges organized in the five Action Areas, are supported by an increasingly comprehensive body of evidence. For example, research findings continue to support the use of social protection floors and systems over the life course, including social protection benefits (e.g., cash transfers) for children, mothers, the unemployed, occupational injury victims, and older persons (e.g., [5–11]). Another example is evidence for the effectiveness of early childhood interventions in improving cognitive development, health service use, and health outcomes in both childhood and adulthood [12–14]. Moreover, novel approaches in systems theory and causal inference used to evaluate social interventions continue to produce evidence of their beneficial effects on community and population health equity [15, 16].

Health sector monitoring of population health inequities within countries has improved at the global level, including WHO’s Health Equity Monitor [17], the Millennium Development Goals [18], and the progressive realization of the SDGs [19]. The importance of implementation of SDH interventions in the SDG era is gaining recognition [20]. However, specific efforts focused on monitoring SDH actions have only recently received attention, via individual national government commitments and of mandated intergovernmental organizations [16, 21–24]. A need persists for monitoring SDH actions to align them with their actors, and not just the situation of SDH themselves (where these cannot be aligned with policy responsibilities) without overburdening existing monitoring systems.

Through the Rio Political Declaration and other WHO resolutions (e.g., WHA62.14), WHO has committed to developing a global monitoring system for action on SDH [25, 26]. The goals of the proposed WHO monitoring system are to (1) track the progressive realization of action on SDH through implementation of the Rio Political Declaration at national and international levels and (2) guide continuous improvement in SDH action by UN Member States and within the UN system by providing regular reports about the status of and trends in such action. The WHO global monitoring system for action on the SDH will complement WHO’s existing tracking of key social and environmental determinants of health. That system is analogous to other WHO monitoring systems focused on monitoring financing and implementation for water and sanitation [27] or implementation of the WHO Framework Convention on Tobacco Control [28].

The first step in establishing the SDH action monitoring system is identifying its domains, which are the five Action Areas of the Rio Political Declaration. SDH action is defined in this context as a human rights, governance, policy, or programmatic intervention that improves health. The second step is identifying relevant measurement concepts (i.e., actions that can be defined, conceptualized, and measured). Ideally, each measurement concept already has standard action indicators that are internationally harmonized, meet minimum quality standards, are based on data available for a majority of UN Member States, and are relevant for key stakeholders (e.g., national governments, international organizations, and civil society). SDH action indicators are performance indicators for inputs, outputs, and outcomes of relevant government interventions (e.g., existence of or coverage with laws, policies, or programs). These indicators may measure SDH action by (1) human rights frameworks; (2) governance structures and mechanisms; (3) social policies and programs; and (4) environmental policies and programs [29]. A recent global stocktake identified SDH-focused monitoring systems reporting action indicators in 16 countries and in five regional and global systems (mostly of WHO), although reporting of relevant indicators to the Rio Political Declaration is limited [29]. Canada was among the first countries to monitor SDH action and has qualitatively reported on national actions to advance the five Action Areas of the Rio Political Declaration [21, 22, 30].

We report in this paper on the process undertaken to (1) identify key measurement concepts for each of the five Action Areas of the Rio Political Declaration; (2) identify suitable candidate SDH action indicators and data sources; and (3) propose a core set of the 15–20 selected indicators, considering the needs of key indicator users, including national governments, international organizations, and civil society, which we propose form part of the initial WHO global system for monitoring action regarding SDH. These tasks were accomplished by an interdisciplinary working group (WG) established by WHO and its Canadian partners, the Public Health Agency of Canada (PHAC), and the Canadian Institutes of Health Research—Institute of Population and Public Health (CIHR–IPPH).

## Methods

The WG implemented processes for identifying a set of indicators that were aligned with measurement concepts drawn from the Rio Political Declaration [3], had high content validity, and relied on available country-specific monitoring data. The Rio Political Declaration with 50 specific actions built directly on and aligned with the recommendations of the Commission on Social Determinants of Health [1]. Therefore, monitoring implementation of actions taken in response to the organizing policy framework of the Rio Political Declaration is in harmony with the long-term efforts on SDH that Member States have pursued.

Recruitment of WG members was a deliberate and extensive process that sought to bring together those with both technical and policy expertise, and with representation from all six WHO regions. This step was important for reflecting considerations for monitoring in the countries of their particular region. The WG comprised 18 experts, including academics, policy-makers from high-, middle-, and low-income Member States; and representatives from WHO. WG members represented Australia, Brazil, Canada, Chile, India, Italy, Kenya, Morocco, Norway, Rwanda, Switzerland, and the USA. The WG also included persons with expertise in indicator development. This broad representation enabled WG members to identify technically sound, feasible, and acceptable candidate indicators while considering the needs of key users of the indicators, including national governments, international organizations, and civil society. The WG was chaired by Patricia O’Campo PhD, University of Toronto, who is a world leader in the study of SDH. The WG was also supported by a secretariat of four graduate students who screened candidate indicators, drafted documents, and provided administrative support during WG meetings.

During a rapid 4-month period, the expert WG members:

1. identified and prioritized key measurement themes and specific measurement concepts related to action on the SDH in the Rio Political Declaration;
2. identified and selected an initial list of potentially suitable candidate indicators for each measurement concept from multiple databases and systems, including the SDG monitoring system; and
3. applied selection and quality criteria for prioritizing a core set of indicators.

**Fig. 1:**
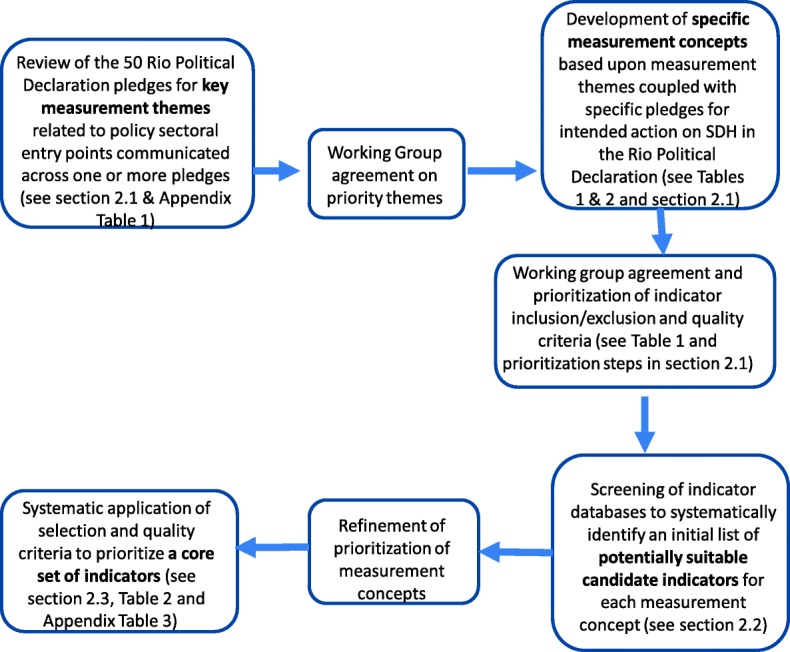
Flow chart describing the expert working group process for arriving at a core set of proposed indicators for measuring social determinants of health (SDH) action

A flow chart of the full process is depicted in Fig. 1. Key terms for the process were also defined early (Table 2).

**Table 2:** Glossary of terms reflecting the components of the social determinants of health (SDH) action monitoring system and corresponding components of the Rio Political Declaration (from narrow to broad)

Component: SDH action monitoring system		Corresponding component: Rio Political Declaration	
Term	Definition	Term	Definition
Domain	Set of measurement concepts that are heuristically related to one another. Five domains are included in the monitoring system that correspond to the five Action Areas of the Rio Political Declaration.	Action Area	A set of related actions in the Rio Political Declaration aimed at enhancing or reorienting capacities of governments or inter-governmental organizations to address the SDH. A total of five (1 to 5) Action Areas of the Rio Political Declaration addresses SDH.
Measurement concept	A defined, measurable aspect of an SDH intervention theme. Themes captured the intervention focus on a pledge or set of pledges, e.g., build social protection floors. A total of 23 measurement themes were proposed, which after debate and refinement, led to the proposal of a final list of 17 measurement concepts.	Pledge	An intended action on SDH belonging to one of five action areas pledged by United Nation Member States. Fifty pledges were included in the Rio Political Declaration. Pledges were enumerated by Roman numerals but several pledges could relate to a common SDH intervention theme.
SDH action indicator	A valid, reliable gauge of the measurement concept that describes the action on SDH.	Action on SDH (“SDH action”)	A determinants-oriented, non-medical governance, policy, or programmatic intervention that improves health equity.

### Identification of measurement themes and measurement concepts derived from the Rio political declaration action areas and pledges

The WG members reviewed each pledge of the Rio Political Declaration to identify and record the frequency of occurrence of themes related to policy sectoral entry points (e.g., social service and protection policies, development strategies, policies, and rights) or such special population groups such as children; women; indigenous populations; informal workers; and lesbian, gay, bisexual, transgender, and intersex (LGBTI) populations. These entry points formed measurement themes, which were defined to ensure a common understanding of the theme across the WG. Broadly, measurement themes were general ideas that might have been reflected in more than one Rio Political Declaration Action Area or pledge (see glossary Table 2). For example, the measurement theme “build social protection floors” referred to “the extent to which governments provide essential health and economic security to populations in need” (see Table 4 in Appendix) and was linked to pledges in three of the Rio Action Areas: (1) to adopt better governance for health and development (pledge 1.x, promote and strengthen universal access to social services and social protection floors); (2) to further reorient the health sector towards reducing health inequities (pledge 3.iv, build, strengthen and maintain health financing and risk pooling systems and prevent persons from becoming impoverished when they seek medical treatment); and (3) to strengthen global governance and collaboration (pledge 4.ii. support social protection floors as defined by countries to address their specific needs and the ongoing work on social protection within the UN systems, including the work of the International Labour Organization, see Table 4 in Appendix for a list and description of measurement themes by Rio Action Area).

These measurement themes, either in isolation or coupled with back-reference to Rio Political Declaration pledges, guided the formation of the more actionable measurement concepts to facilitate the proposal of suitable indicators of SDH action. Building on the measurement theme related to social protection discussed in an earlier section of the paper, the measurement concept of level of public social protection was identified. Other examples of measurement concepts included level of implementation of mechanisms for participation of civil society; provision of public laws guaranteeing workers human rights for informal workers; level of implementation of mechanisms for ensuring integration of equity into health systems, policies and programs; and North–South, South–South sharing to develop holistic policies addressing inequalities and sustainable development.

The WG members engaged in an iterative process for refining and prioritizing relevant measurement concepts for action on SDH, as follows. Measurement concepts that were mentioned more frequently within or across domains were prioritized over those mentioned less frequently. Preference was given to measurement concepts unique to action regarding SDH and well supported by evidence as recommended by the WG members. Measurement concepts were developed with attention paid to their relevance/applicability across different country contexts. Prioritized measurement concepts also aimed to reflect a balance of indicators for SDH-focused human rights or governance structures or mechanisms and policies or programs on specific determinants of health. Furnishing these discussions with information on data availability by country as described in the next section also shaped the finalization and definition of measurement concepts.

### Identification and selection of the most suitable indicators for each measurement concept from multiple databases or systems

We screened existing reports and international databases proposed by WG members and external academic experts that are used for monitoring by international organizations or academic institutions. A prioritized source for indicators was the monitoring system of the SDGs with its 261 indicators [31] because using SDG indicators was regarded as crucial for ensuring alignment of the SDH action monitoring system with the 2015–2030 SDG agenda [32]. Indicators proposed by WHO and the World Bank for universal health coverage were also prioritized to ensure alignment with the universal health coverage agenda [33–35]. In total, we screened 21 data sources and references produced by the International Labour Organization, the World Bank, the Pan American Health Organization, and others (see Table 5 in Appendix for a list of sources consulted).

To ensure consistency with best practices of indicator selection, we reviewed existing relevant reports of international monitoring efforts as well as the indicator development literature [21, 36–41]. This process guided criteria development for arriving at the core set of proposed indicators, including inclusion or exclusion criteria of screened indicators for an initial list of candidate indicators, and a set of quality criteria for evaluating candidate indicators to form the core set of proposed indicators (described in the next section).

#### Inclusion and exclusion criteria

To be eligible for inclusion in the initial list, indicators were required to

1. capture a key measurement concept;
2. measure an action (or intervention) regarding SDH, defined as
  - a governance intervention focused on SDH or health equity;
  - a social intervention that improves health or health equity; or
  - an environmental intervention that improves SDH or health equity [29];
3. measure a modifiable action [29, 40];
4. measure a national-level government action (Rio Political Declaration Action Areas 1, 2, 3 and 5) or a global-level action from the countries’ government or international governmental organizations (Rio Political Declaration Action Area 4); and
5. be a quantitative or, if qualitative, at a minimum be a categorical indicator (i.e., existence of laws) [21, 40].

An indicator was excluded from the initial list if it:

1. does not measure an action regarding SDH (i.e., measures an intention as opposed to an action);
2. has no data available or has data becoming available only over the long term (> 3 years);
3. measures an action that cannot be modified; or
4. measures an action taken at the local level only.

The WG debated whether to include only continuous quantitative indicators focusing on the effectiveness of interventions (e.g., coverage), but this criterion was relaxed slightly on the basis of the nature of the measurement concepts as well as data availability constraints.

To further reduce the list of candidate indicators that met the inclusion criteria, we assessed the level of alignment with the measurement concepts. WG members scored each indicator on the measurement concept match criteria, where a score of 1 was assigned when the indicator was not at all in accordance with the corresponding measurement concept, and a score of 7 was assigned when it was completely in accordance with the corresponding measurement concept. A rationale for the score was also provided. Table 6 in Appendix provides detailed examples of candidate indicators that met inclusion criteria for measurement concepts as well as measurement concept alignment scores for domains 1–3.

### Application of quality assessment criteria to arrive at a core set of indicators

The initial list of indicators was put forward on the basis of face validity and technical quality. In the process of finalizing the proposal for the monitoring framework the WG members developed and applied a broad set of technical criteria to apply to the indicators. Several of the criteria could be operationalized and formed part of formal documented assessments as described in Table 6 in Appendix. The technical quality assessment criteria were defined and applied as follows:

1. For harmonization purposes, preference was given to indicators from the SDG indicator system and concepts covered in SDG targets [42].
2. Indicators that could demonstrate change over time and were feasible were preferred. This meant preference was given to indicators with regular reporting of at least 5-year intervals, but greater preference was given to indicators with 2–3-year reporting intervals.
3. Indicators should demonstrate benefits for SDH, health service use, health outcomes, or health equity. Although no extensive or formal assessment of studies on the effectiveness of government actions was conducted, where WG members had knowledge of studies describing the links between social determinants and the indicators, the information was noted. Consequently, preference was also given to indicators measuring actions with a stronger evidence base over those with a weaker evidence base [21].
4. Expected acceptability (i.e., user-inspired, responding to data needs, and acceptable to such key stakeholders as national governments or civil society) [38, 41]. The assessment of these criteria was based on the knowledge and background of the WG members, who had been nominated by the WHO regional offices to represent the experiences of the countries of their particular region.
5. Feasibility and cost-effectiveness of providing the information—whether easy to obtain without additional burden for the producer or guardian of the data [38, 41]. Preference was given to official data over other sources because this would enable WHO and other countries to gather data more cost-effectively.
6. Type—preference was given to continuous indicators (e.g., the proportion of a population covered by a social protection floor) over ordinal indicators (e.g., provision of social protection floors was complete versus medium versus low) over binary indicators (e.g., social protection floor was provided versus not provided).
7. Applicability across diverse country contexts and global harmonization [38].
8. SMART criteria [39], as follows:
  **S**pecificity—targets a specific area for improvement; **M**easurability—is easy to measure, interpret, and communicate with straightforward policy implications (avoid composite indices); **A**ssignability—clearly specifies who will take the action; **R**ealistic—an action that realistically can be taken, given available resources; and **T**ime-relatedness—an action that can be changed over time (e.g., annual changes are feasible). The SMART criteria overlap with some of the criteria described previously, but the WG members believed that making explicit reference to them was important because they are commonly used in indicator development work.

On the basis of the number of technical quality assessment criteria met, the indicator was given a rating on a scale of 1–8 and a rationale for the score.

This quality review also included an assessment of the indicator’s methodologic development and data availability as follows:

- Tier 1—Represented an indicator that is conceptually clear with established methodology and standards to generate the indicator and data regularly produced by countries.
- Tier 2—Indicators were conceptually clear on the basis of established methodology and standards but where data were not regularly available across countries.
- Tier 3—Indicators were not based on established methodology or standards.

Table 6 in Appendix provides detailed examples of candidate indicators and the quality and technical ratings and overall assessment of the availability of indicators by domain.

## Results

The WG members developed and prioritized 23 measurement themes, and 19 measurement concepts. Following the iterative process of evaluation, we identified a core set of 36 candidate indicators for the monitoring system on SDH action.

The core set of indicators proposed by the WG is presented in Table 3. Each indicator was uniquely named, with the nomenclature reflecting the indictor’s domain, measurement concept (that at times included an indication of being an equity measure), and the individual indicator. For example, Indicator 3.1I.1 Parity index (by wealth quintile) in coverage with safely managed drinking water is:

- an indicator from domain 3 (i.e., **3**.1I.1)
- the measurement concept 1I (i.e., 3.**1I**.1), where the “I” indicates that inequality in an intervention is measured; and
- the first indicator for this measurement concept (i.e., 3.1I.**1**).

**Table 3:** Final core set of 36 indicators and sources of data proposed by the working group to consider for the monitoring system on taking action regarding social determinants of health (SDH)

Domain/Measurement concept	Indicator
Domain 1: National governance	
1.1 Level of public social protection	1.1.1 Percentage of the population covered by social protection floors/systems below the poverty line [SDG Indicator 1.3.1]
1.1I Gender inequities in the level of public social protection	1.1I.1 Parity index (female/male) for the percentage of the population covered by social protection floors/systems below the poverty line [SDG Indicator 1.3.1, disaggregated data]
1.2 Level of public provision of early childhood education	1.2.1 Participation rate in organized learning (one year before the official primary entry age) [SDG Indicator 4.2.2]
1.2I Gender inequities in the level of public social protection	1.2I.1 Parity index (female/male) for participation rate in organized learning (one year before the official primary entry age) [SDG Indicator 4.2.2, disaggregated data]
1.2II Income inequities in the level of public social protection	1.2II.1 Parity index (bottom/top wealth quintile) for participation rate in organized learning (one year before the official primary entry age) [SDG Indicator 4.2.2, disaggregated data]
1.a Provision of the rights and public laws guaranteeing self-determination of indigenous peoples	[no indicator yet identified]
1.b Provision of public laws guaranteeing human rights for transgender populations	1.b.1 Presence or lack of laws that criminalize transgender identity and expression, protect against discrimination on the basis of gender identity/gender expression as a category, and determine the legal right for individuals to determine their legal gender and name^a,^* [United Nations Development Programme]
1.c Provision of public laws guaranteeing human rights for sex workers	1.c.1 Presence or lack of laws that criminalize sex work and protect the public health of sex workers* [Review of national legislation]
1.d Provision of public laws guaranteeing workers human rights for informal work	1.d.1 Increase in national compliance of labor rights (freedom of association and collective bargaining) based on International Labour Organization textual sources and national legislation [SDG Indicator 8.8.2]
1.e Level of intersectoral action for health and health equity	1.e.1 Whether a national policy exists that addresses at least two priority determinants of health amongst target populations^b^* [Pan American Health Organization (PAHO)]
Domain 2: Participation	
2.a Mechanisms for guaranteeing transparency in policymaking	2.a.1 Whether country has adopted and implemented constitutional, statutory, or policy guarantees for public access to information [SDG Indicator 16.10.2]
2.b Level of implementation of mechanisms for participation of civil society	2.b.1 Whether the country has accountability mechanisms that support civil society engagement in health impact decisions* [PAHO]
	2.b.2 Whether mechanisms exist to engage communities and civil society in the policy development process across all sectors* [PAHO]
2.c Level of implementation of mechanisms for participation of civil society in policymaking for indigenous peoples	2.c.1 Number of policies that recognize the duty to consult and cooperate in good faith with indigenous peoples to obtain their free, prior and informed consent before adopting and implementing legislative or administrative measures that may affect them [World Conference on Indigenous Peoples commitment, paragraph 3*]
	2.c.2 (1) Existence of special measures to strengthen capacity of indigenous peoples’ representative institutions; (2) existence and capacity of national human rights institutions to reach out to vulnerable groups such as indigenous peoples; (3) institutional mechanisms and procedures for consultation with indigenous peoples, in accordance with international standards* [UN Declaration on the Rights of Indigenous Peoples]
	2.c.3 (1) Provisions for direct participation of indigenous peoples’ elected representatives in legislative and elected bodies; (2) recognition in the national legal framework of the duty to consult with indigenous peoples before adopting or implementing legislative or administrative measures that may affect them* [United Nations (UN) Declaration on the Rights of Indigenous Peoples]
2.d Level of implementation of mechanisms for participation of civil society in policymaking for transgender populations	2.d.1 Presence/lack of laws that prohibit lesbian, gay, bisexual, transgender, and intersex persons from forming organizations and participating in political parties and social movements* [United Nations Development Programme]
Domain 3: Health sector reorientation	
3.1 The level of comprehensive, equitable basic service coverage by health systems (including primary health care and the right to health)	3.1.1 Percentage of population using safely managed drinking-water services [SDG Indicator 6.1.1]
	3.1.2 General government expenditure on primary health care and health promotion as a proportion of general government expenditure (proxy, if data are unavailable: 3.1.2 General government expenditure on health as a proportion of general government expenditure) [WHO]
3.1I Inequities in the level of comprehensive, equitable basic service coverage by health systems (including primary health care and the right to health)	3.1I.1 Parity index (by wealth quintile) in coverage with safely managed drinking water [SDG Indicator 6.1.1, disaggregated data]
3.2 Level of financial health protection	3.2.1 Percentage of population with catastrophic health expenditure (universal health coverage) [WHO]
3.2I Inequities in level of financial health protection	3.2I.1 Out-of-pocket (OOP) payments as % of income amongst lowest wealth quintile or OOP as % of income amongst highest wealth quintile [WHO, disaggregated data]
3.3 Level of integration of equity into health systems, policies and programmes	3.3.1 Percentage of total government health expenditure on prevention and public health services [Organization for Economic Co-operation and Development health accounts; WHO national health accounts]
	3.3.2. Equity-adjusted universal health service coverage index* [WHO]
3.a Mechanisms for ensuring integration of equity into health systems, policies and programmes	3.a.1 Existence of policies and strategies to address health inequalities and social determinants of health (Existence of a national policy that supports routine consideration of health equity in health promotion and disease prevention programs) [World Health Organization European Region (EURO)]
	3.a.2 Elements in national policies to address health inequities and social determinants of health (Existence of a national policy that supports routine consideration of health equity in health promotion and disease prevention programs) [EURO]
Domain 4: Global governance	
4.1 Level of international funding for comprehensive, equitable basic service coverage by health systems (including primary health care and the right to health)	4.1.1 Amount of water- and sanitation-related official development assistance that is part of a government coordinated spending plan [SDG Indicator 6.a.1]
4.a Level of implementation of international agreements that improve the SDH	4.a.1 The country’s performance on the International Health Regulations capacity and health emergency preparedness index [SDG Indicator 3.d.1]
	4.a.2 Number of countries with tax policies that have been implemented to reduce tobacco demand [World Health Organization Framework Convention on Tobacco Control]
4.b Participation of developing countries in international policymaking	4.b.1 Percentage of members or voting rights of developing countries in international organizations [SDG Indicator 10.6.1/16.8.1]
4.c North-South, South-South sharing to develop holistic policies addressing inequities and sustainable development	4.c.1 US dollar value of financial and technical assistance (including through North-South, South-South and triangular cooperation) committed to developing countries [SDG 17.9.1]
Domain 5: Monitoring and accountability	
5.1 Disaggregation of health data according to SDH	5.1.1 Percentage of indicators in the Global Health Observatory that are provided and disaggregated by a social characteristic [WHO]
5.a. Level of implementation of SDH-focused monitoring systems	5.a.1 Country has dedicated SDH action monitoring system (as per WHO definition to be developed)* [WHO/PAHO]
	5.a.2 Country has dedicated monitoring system for health inequalities [WHO]
5.b. Financial investment in research and evaluations of SDH interventions to promote equity	5.b.1 Proportion of national health research spending related to actions on SDH* [Canadian Institutes of Health Research—Institute of Population and Public Health]
5.c. Mechanism for guaranteeing access to information as a key component of research, monitoring and evaluations to ensure accountability and justice	5.c.1 Whether country has adopted and implemented constitutional, statutory or policy guarantees for public access to information [SDG Indicator 16.10.2]

Key: Governance interventions (or processes) are indicated with a lowercase letter (e.g., 3.a.1 measurement). A capital Roman numeral I or II refers to indicators measuring inequities in the population coverage with an intervention (e.g., 3.1I.1) (mainly parity indices [ratio of disadvantaged to advantaged population in intervention coverage])

*Indicator does not have comprehensive data availability (i.e., does not have all of: established methods, international standards, and data available across many countries)

^a^Composite index composed from three individual binary indicators

^b^A composite index could be composed of this indicator and additional binary indicators from the Pan American Health Organization’s Health in All Policies regional monitoring system

Five indicators of the core set expressly deal with inequities in intervention coverage, 21 cover governance interventions, and the remaining 10 address social and environmental interventions to improve the SDH.

Tables in the appendices provide information about the results of the development of measurement themes and concepts (Table 4 in Appendix) as well as information about the sources of data consulted to identify the candidate indicators (Table 5 in Appendix). An example of candidate indicators for consideration for the measurement concepts and their levels of alignment, face validity, and technical quality are presented in Table 6 in Appendix. What is not shown in Table 6 in Appendix are measurement concepts for which we could not find relevant indicators. For example, for the following measurement concepts, we were unable to locate candidate indicators: measure extent to which equity impacts of all government policies assessed routinely in decision making (governance); provision public laws guaranteeing self-determination of Indigenous peoples (governance); promote platforms for knowledge exchange of equity-oriented good practices and successful experiences (health sector reorientation); represent level of implementation of international agreements that improve the SDH (global governance); and ensure that justice and accountability are key components of research and evaluations (monitoring and accountability).

## Discussion

### Summary of findings

We present the methodology used to identify and prioritize key measurement concepts from the Rio Political Declaration and to select relevant, high quality indicators with real-life restrictions on data availability to form the first proposed core set of indicators to guide global monitoring for action on SDH. This set of indicators was presented to a large group of UN Member States and global technical experts at the International Technical Meeting on Measuring and Monitoring Action on the Social Determinants of Health, which took place in Ottawa, Canada, in June 2016 [43] to further guide a broader SDH Action Monitoring System for Member Countries. This is an innovative initiative as previous literature focused explicitly on indicators of determinant vs policy outcomes [34, 44]. Ultimately, this set of indicators will enable policy-makers to track progress in addressing SDH and to build the evidence base of effective actions for reducing health inequities (e.g., [5–9, 23, 45]). Recent evidence emphasizes the importance of cross-government commitment to addressing the root of SDH inequities [33]. Principles guiding identification of the indicator set included input from a diverse working group, attention to equity in the concepts and indicators, and reliance on existing indicators.

### Lessons learnt for global monitoring system development

We identified several strengths of our methods for developing a proposed core set of indicators. First, our WG members included diverse technical and policy experts from all six WHO regions. This broad representation enabled the WG to identify technically sound, feasible, and acceptable candidate indicators while considering the needs of key users of the indicators. Second, we drew on best practices for indicator selection [21, 38–41] in developing the inclusion or exclusion and quality assessment criteria applied to candidate indicators during the screening process. We also adopted an iterative approach that involved consideration of data availability to refine and prioritize measurement concepts.

A guiding principle for the indicator set development was a strong focus on equity. First, some disaggregated indicators (i.e., by sex, age, income, etc.) were included where the concept and data permitted. As is becoming the reference standard in the SDG era with its commitment of leaving no one behind [32], disaggregated indicators better capture how action on the SDH works or does not work to reduce health inequities. To further incorporate inequity measurement into the monitoring system, the WG sought to incorporate indicators that would focus specifically on actions directly affecting the most vulnerable groups, thereby ensuring that no one is left behind. This included indicators that measure inequities experienced by indigenous peoples, children, women, persons living in poverty, LGBTI populations, and informal workers.

We identified six key challenges in the development of the core set of indicators. First, we discovered that indicators monitoring SDH-focused interventions are still not routinely collected for certain areas. For example, indicators for intersectoral actions (e.g., Health in All Policies) have only recently been assessed qualitatively by PAHO [34]. Indicators related to the health expenditures on health promotion are focused only in Organization for Economic Cooperation and Development countries. The WG members therefore relied heavily on the SDG indicator system, which presents a number of novel SDH action indicators.

Second, indicators that are explicitly focused on equity are limited. An equity orientation to monitoring health determinants is a new area of focus for health surveillance and monitoring systems [46], although examples such as indicators for measuring actions related to multisectoral governance and participation are starting to emerge [40]. Moreover, although many populations deserving additional attention (e.g., indigenous peoples, children) during monitoring efforts were considered, our final indicators were limited in number so some such as migrants or persons with disabilities were not reflected in our final set. Similarly, another example of a trade-off was around exclusion of informal payments (tipping or bribes) in health systems from the final set limiting the representation of the domain measurement concept “mechanisms for ensuring integration of equity into health systems, policies and progammes” (Table 3, item 3.a).

Third, although we sought to apply the quality assessment criteria with care, time constraints and limited resources presented some challenges. For example, one criterion required that we give priority to indicators with stronger existing evidence about the benefits of that particular SDH. The application of the criterion relied on the expert panel’s knowledge base; neither time nor resources allowed more extensive literature reviews of this particular topic. Another area affected by our short time frame, and will have to be examined in future work in this area, was our inability to examine and identify process indicators that reflect the dynamic nature of and interaction between two or more measurement concepts or domains.

A fourth challenge identified was identifying how to capture the way interventions are implemented and in what types of contexts including such questions as those pertaining to Indigenous populations, that in some countries are regional concerns and not applicable to the country as a whole. Multiple SDH indicators measure actions taken to improve SDH, but they are not designed to capture the degree of implementation or coverage or whether the action was successful. The political, social, cultural, or community contexts in which the interventions are implemented are not always clear from the indicators. This is important information for determining if or how an intervention works or does not work.

Fifth, assessing the effectiveness and cost-effectiveness of SDH-focused interventions is difficult on the basis of indicators alone. The range of information on cost-effectiveness is limited by the extent of effectiveness studies, which are inherently challenged by the complexity of the SDH interventions [47]. Consolidating the evidence base of countries’ implementations of complex policy-level interventions will be necessary for ensuring that the monitoring system is able to capture SDH actions that are considered best practices. Although systematic reviews are useful for building the evidence base for a single sector, especially health sector initiatives, other methods of summarizing the effective implementation and impacts of complex cross-sectoral policies will likely be required [48, 49].

Finally, the WG also experienced limitations in the available data sources. For instance, the domains of the monitoring system capture all Rio Political Declaration Action Areas, but the scan of globally available indicators revealed a disproportionate number of existing indicators for each Action Area. Action Area 4 strengthen global governance and collaboration for addressing the SDH (domain 4) and Action Area 5 monitor progress and increase accountability (domain 5) had only six and five candidate indicators, respectively. And while data availability was a criterion, conceptual fit and feasibility for data collection were also important considerations. For example, indicator 5.b.1 was only available for Canada but this was an indication of the feasibility of developing and reporting a standard metric. Additional work is needed to develop more suitable indicators to capture action to strengthen global governance and collaboration, and action for monitoring progress and increasing accountability.

### Implications for research and practice

More work will be needed as the monitoring system continues to develop. Where data gaps have been identified, further exploration of existing data sources and potential new sources will be necessary. A need also exists for evaluating the monitoring system for relevance, accuracy, and validity, and potentially for adapting indicators as new data sources become available or data quality improves. More research on under-studied but crucial interventions on SDH is needed (e.g., mechanisms and structures for intersectoral action for health and global health governance interventions that protect health and health equity from international trade agreements) to further strengthen the evidence base for SDH action indicators, which might necessitate developing novel research methods.

## Conclusions

Because of the pervasive and growing inequalities worldwide, there is an emerging trend towards promoting and monitoring government action on SDH. Yet few existing indicators adequately capture a government’s intent to and implementation of policies and programs to address SDH. Many challenges exist to developing such indicators, some of which have been described here (e.g., those related to data availability). While the methods presented in this paper extend the state of the art in measuring government action on SDH, future efforts should attend to the existing gaps to create a strong set of indicators for monitoring SDH in high- and low-income countries alike.

## Appendix 1

**Table 4:** Measurement themes identified in the Rio Political Declaration on the Social Determinants of Health (SDH) regarding policy sector entry points or special population groups

Measurement theme	Description of measurement theme
Governance	
Build social protection floors	The extent to which governments provide essential health and economic security to populations in need
Early childhood development and education	The extent to which governments support healthy development and equitable education for children
Supporting healthy work or workers	The extent to which governments support healthy workplaces, or occupational health and safety
Healthy public policy or health in all policies	The extent to which governments consider impacts of policies (both within and outside the healthcare sector) on population health and the healthcare system
Knowledge transfer (health impact assessment)	The extent to which governments disseminate and otherwise make available results of health impact assessment to all stakeholders
Role of stakeholders considered	The extent to which governments take into account and consult with all stakeholders, including those in the public and private sectors, as well as those in the community
Inclusive policy or vulnerable populations targeted	The extent to which governments produce policy that is tailored for vulnerable populations and populations in need
Intersectoral collaboration	The extent to which divisions in the government work together to produce multi-sectoral policies and programs
Structural determinants of health	The extent to which governments support the equitable improvement of structural determinants of health
Participation	
Public participation	Government that encourages and facilitates public participation in policy development and decision-making
Inclusive government	Government that encourages and facilitates participation of all stakeholders in policy development and decision-making
Accountability	Government that acknowledges and responds to public questions and concerns about policy development and decision-making
Transparency	Government that is clear and open with the public about policy development and decision-making process and outcomes
Open information	Government that is transparent and forthcoming with information about policy development and decision-making process and outcomes
Indigenous peoples	Government that encourages and facilitates meaningful participation of indigenous peoples in policy development and decision-making
Health sector reorientation	
Social protection	The extent to which the healthcare sector (and global governance sector) provides essential services to populations in need
Access to medicine and healthcare	The extent to which healthcare services and medicines are equitably accessible across populations
Equity in health systems, policies, and programs (or equity measures integrated in policy processes)	The extent to which systems, policies, and programs confer equitable health benefits across all populations
Health equity impact assessment (or equity impacts of policies)	The extent to which equity in health benefits are measured and reported for systems, policies, and programs in the healthcare sector
Global governance	
Implement international policy or declaration (with caveat that more than signing of declaration needs to be captured)	Tangible evidence that international policies and declarations are implemented in countries that are signatories.
Monitoring and accountability	
Data availability, and routine disaggregation of health data	Availability of high-quality, routinely collected and disaggregated data regarding population health for monitoring progress
Promotion and investments in research	Financial interest and support for research that measures and monitors SDH
Evaluation of program impacts and attention to health equity outcomes	Research and evaluation of health and equity impacts of programs and policies

## Appendix 2

**Table 5:** Selection of sources screened for identifying indicators to measure government action on the social determinants of health, per the Rio Political Declaration

Organization/Institution/Data Steward	Report or database title	URL for website or PDF
1. Center for Economic and Social Rights	CESR Human Rights Policy Brief: The Measure of Progress: How Human Rights Should Inform the Sustainable Development Goals Indicators	http://www.cesr.org/measure-of-progress
2. World Health Organization	Measuring and monitoring intersectoral factors influencing equity in universal health coverage (UHC) and health Summary report of a meeting in Bellagio, 6 - 8 May 2014	http://www.who.int/social_determinants/events/Meeting_Bellagio_May2014_Report_FINAL_for_web_11_Jul.pdf
3. Indigenous Peoples Major Group	Policy Brief on Sustainable Development Goals and post-2015 Development Agenda: A Working Draft	https://sustainabledevelopment.un.org/content/documents/6797IPMG%20Policy%20Brief%20Working%20Draft%202015.pdf
4. Institute for Democracy and Electoral Assistance	Global State of Democracy Indices	https://www.idea.int/data-tools/tools/global-state-democracy-indices
5. MACHEquity	MACHEquity Data Center	http://machequity.com/
6. Pan American Health Organization	(Draft Document) Plan of Action on Health in all Policies: Validation of Implementation Indicators, 2015	http://www.paho.org/hq/index.php?option=com_docman&task=doc_download&gid=29647&Itemid=270&lang=en
7. Social Security Administration (United States)	Social Security Programs Throughout the World	https://www.ssa.gov/policy/docs/progdesc/ssptw/
8. United Nations Water	Metadata on Suggested Indicators for Global Monitoring of SDG 6 on Water and Sanitation	http://www.unwater.org/publications/monitoring-water-sanitation-2030-agenda-sustainable-development-executive-briefing-2/
9. United Nations	Compilation of Metadata Received on Indicators for Global Monitoring of the Sustainable Development Goals and Targets	http://unstats.un.org/sdgs/files/Metadata%20Compilation%20for%20SDG%20Indicators%2023%20October%202015%20Update.pdf
10. United Nations Environment Programme	Environmental Data Explorer, Core Indicators	http://geodata.grid.unep.ch/extras/indicators.php
11. United Nations, Department of Economic and Social Affairs, Statistics Division	Sustainable Development Goal Indicators Official list of Sustainable Development Goal Indicators	http://unstats.un.org/sdgs/indicators/indicators-list/
12. University of Gothenburg, Quality of Government Institute	QoG Standard Database: Quality of Government, Civil Society/Population/Culture, Education, Energy and Infrastructure, Health, Labour Market, Political System, Welfare	https://www.qogdata.pol.gu.se/dataarchive/qog_bas_jan16.pdf
13. WHO Framework Convention on Tobacco Control, World Health Organization	2014 Global Progress Reports on Implementation of the WHO Framework Convention on Tobacco Control	http://www.who.int/fctc/reporting/2014globalprogressreport.pdf
14. World Bank Group	Worldwide Governance Indicators	http://data.worldbank.org/data-catalog/worldwide-governance-indicators
15. World Bank Group	Women, Business and the Law 2016: Getting to Equal	http://documents.worldbank.org/curated/en/455971467992805787/Women-business-and-the-law-2016-getting-to-equal
16. World Economic Forum	Global Gender Gap Report 2015	https://www.weforum.org/reports/global-gender-gap-report-2015
17. World Health Organization	Monitoring the Building Blocks of Health Systems: A Handbook of Indicators and Their Measurement Strategies, 2010	https://www.who.int/healthinfo/systems/monitoring/en/
18. World Health Organization, UN Water	UN-Water Global Analysis and Assessment of Sanitation and Drinking-Water (GLAAS) 2014: Investing in Water and Sanitation: Increasing Access and Reducing Inequalities	http://www.who.int/water_sanitation_health/publications/glaas_report_2014/en/
19. World Policy Analysis Center at the University of California Los Angeles	WORLD’s Areas Public Use Data	http://worldpolicycenter.org/
20. World Values Survey	Online Data Analysis	http://www.worldvaluessurvey.org/WVSContents.jsp

## Appendix 3

**Table 6:** Measurement concepts along with the initial candidate list of indicators presented by domain. Assessments of quality and measurement concept alignments are also presented for each indicator and domain

Measurement concept	Candidate indicator	Tier	Quality assessment
Domain 1: Governance			
1.1 Level of intersectoral collaboration for health and health equity	1.1.1 National or subnational policy addressing the reduction of health inequities established and documented.	Tier II	1. Measurement concept match ating (3/7). This indicator does not align well with the measurement concept. 2. Technical quality rating: (2/8). This indicator seeks to measure national policies aimed at reducing health inequities. However, this indicator only meets two of the technical quality criteria. Data are currently available for this indicator.
	1.1.2 Whether a national policy exists that addresses at least two priority determinants of health amongst target populations	Tier II	1. Measurement concept match rating (3/7). This indicator does not align well with the measurement concept. 2. Technical quality rating: (4/8). This indicator seeks to measure national policies aimed at reducing health inequities. However, this indicator only meets two of the technical quality criteria. This indicator is also a binary indicator. Data are available for this indicator.
	No candidate indicator captures the measurement concept well, is technically feasible, and has data availability. The opportunity might exist for leveraging off the WHO Regional Office for Europe and the Pan American Health Organization indicators. Investment in the development of a new indicator may be beneficial (e.g., a standard indicator for intersectoral action for health).		
1.2 Level of implementation of health equity impact assessment for relevant government policies	1.2.1 Proportion of seats held by women in (a) regional parliaments and (b) local governments	Sustainable Development Goals (SDG) (a) Tier I, (b) Tier III	1. Measurement concept match rating (4/7). This indicator does not align well with the measurement concept. 2. Technical quality rating: (5/8). This indicator seeks to measure proportion of seats held by women on key decision-making bodies. Whereas this indicator meets most of the technical quality criteria, the unavailability of data at the local government level makes it challenging to recommend this for inclusion. Only the first aspect of this indicator has data readily available.
	The candidate indicator does not capture the measurement concept and does not meet the minimum mark to be included in the monitoring system. Therefore, we recommend that a new indicator be developed.		
1.3 Level of public social protection	1.4.1 Percentage Parity index (female or male) for the percentage of the population covered by social protection floors or systems	SDG Tier I	1. Measurement concept match rating (6/7). This indicator captures the measurement concept. 2. Technical quality rating: (6/8). This indicator aligns with the SDGs and is highly accepted in various countries. Data are readily available for this indicator.
	The candidate indicator captures the measurement concept, if technically feasible, and has data availability. It is suitable for inclusion in the monitoring system. The indicator could be further refined (e.g., could limit it to only the population living in poverty).		
1.4 Gender equity in level of public social protection	1.4.1 Parity index (female or male) for the percentage of the population covered by social protection floors or systems		1. Measurement concept match rating (6/7). This indicator captures the measurement concept. 2. Technical quality rating: (6/8). This indicator aligns with the SDGs and is highly accepted in various countries. Data are readily available for this indicator, and the parity index can be computed.
	The candidate indicator captures the measurement concept, if technically feasible, and has data availability. It is a suitable for inclusion in the monitoring system. The indicator could be further refined (e.g., could limit it to only the population living in poverty).		
1.5 Level of public provision of early childhood education	1.5.1 Participation rate in organized learning (one year before the official primary entry age)	SDG Tier I	1. All three indicators are aMeasurement concept match rating (6/7). This indicator captures the measurement concept. 2. All three indicators are aTechnical quality rating: (6/8). This is an SGD indicator and is accepted in various countries. Data are readily available for this indicator.
	1.5.2 Proportion of schools with access to: (a) electricity; (b) the Internet for pedagogical purposes; (c) computers for pedagogical purposes; (d) adapted infrastructure and materials for students with disabilities; (e) basic drinking water; (f) single-sex basic sanitation facilities; and (g) basic hand washing facilities (as per the WASH indicator definitions)	SDG Tier II	1. Measurement concept match rating (5/7). This indicator captures the measurement concept. 2. Technical quality rating: (6/8). This indicator aligns with the SDGs and is highly accepted in various countries. Data are readily available for this indicator.
	The candidate indicator 6.1 is prioritized over 6.2, because it is a better fit with the measurement concept and has full data availability. The prioritized indicator is fit for purpose and does not require further development.		
1.6 Income equity in level of early childhood education	1.6.1 Parity index (bottom or top wealth quintile) for participation rate in organized learning (one year before the official primary entry age)	SDG Tier I	1. All three indicators are aMeasurement concept match rating (6/7). This indicator does capture the measurement concept. 2. All three indicators are aTechnical quality rating: (7/8). This indicator aligns with the SGDs and is highly accepted in various countries. Data are readily available for this indicator and the parity index can be computed on the basis of the data.
	The candidate indicator captures the measurement concept, if technically feasible, and has data availability. The indicator is fit for purpose and does not require further development.		
1.7 Provision of public laws ensuring human rights	1.7.1 Whether laws and regulations are in place that guarantee women and adolescents access to sexual and reproductive health services, information and education (official records)	SDG Tier III	1. All three indicators are aMeasurement concept match rating (6/7). This indicator captures the measurement concept. 2. All three indicators are aTechnical quality rating: (5/8). This indicator aligns with the SDGs and is highly accepted in various countries. However, this indicator is a binary indicator, but data are not readily available for this indicator.
	1.7.2 Whether a legal framework (including customary law) is in place that guarantees women’s equal rights to land ownership or control	SDG Tier III	1. Measurement concept match rating (6/7). This indicator captures the measurement concept. 2. Technical quality rating: (5/8). This indicator aligns with the SDGs and is highly accepted in various countries. However, this indicator is a binary indicator, but data are not readily available for this indicator.
	1.7.3 Whether legal frameworks are in place to promote equality and non-discrimination on the basis of sex	SDG Tier III	1. Measurement concept match rating (6/7). This indicator captures the measurement concept. 2. Technical quality rating: (5/8). This indicator aligns with the SDGs and is highly accepted in various countries. However, this indicator is a binary indicator, but data are not readily available for this indicator.
	All three indicators are a good match with measurement concepts, but have data availability over the long term only.		
	OVERALL ASSESSMENT DOMAIN 1: Multiple indicators in this domain tap into the prioritized measurement concepts. Most indicators in this domain are also SDG indicators that are collected in different countries. The underlying level of measurement for some indicators in this domain is binary. Efforts should be made to obtain other quantitative indicators. The proposed prioritized indicators capture the measurement concepts moderately well.		
Domain 2: Participation			
2.1 Level of transparency in policymaking	2.1.1 Whether the country has adopted and implemented constitutional, statutory or policy guarantees for public access to information	SDG Tier II	1. Measurement concept match rating (6/7): Public access to information is an integral part of transparency in policymaking. For this reason, the *measurement concept – indicator* match rating was considered moderate to high. This indicator is also well-aligned with Domain 5 (monitoring and accountability). 2. Technical quality rating (5/8): This indicator refers to a specific, measurable government action (criteria 1 and 6) that is applicable across diverse country contexts (criterion 7). Given that the indicator is aligned with the SDGs (criterion 2), it will likely have high acceptability (criterion 8). As Tier II, the data is not readily available (criteria 3 and 4). Further work could be done to transform this binary regional indicator into a national-level indicator (criterion 5).
	2.1.2 Whether the country has systems to track and make public allocations for gender equality and women’s empowerment	SDG Tier III	1. Measurement concept match rating (5/7): This indicator aligns with tracking and sharing information regarding funding for initiatives that address gender equity but this indicator is more specific than 2.1.1. 2. Technical quality rating (4/8): This indicator is aligned with the SDGs (criterion 2), is acceptable (criterion 8), and is applicable across diverse country contexts (criterion 7) and well-defined government action (criteria 1). Given that this indicator is Tier III, information may not be available in the near future and could require data collection.
	2.1.3 Whether country has met their commitments and obligations in transmitting information as required by each relevant agreement on hazardous waste and other chemicals	SDG Tier I	1. Measurement concept match rating (4/7): This indicator addresses transparency in policymaking but it does not specifically measure transparency with the public, which is central for increasing participation as defined in the Rio pledge 2. 2. Technical quality rating (6/8): Given that this indicator is Tier I, data are readily available, routinely collected and used (criteria 2, 4 and 6). It will thus be low or no cost and have high acceptability (criteria 5, 7 and 8).
	The candidate indicator 2.1.1 best captures the measurement concept, is technically feasible, and has some data availability. If data availability is limited, 2.1.3, which is immediately available, could be used as a placeholder indicator, until 2.1.1 becomes available. Co-indicator for Domain 5: monitoring and accountability.		
2.2 Level of implementation of mechanisms for participation of civil society in policymaking	2.2.1 Percentage of local administrative units with established and operational policies and procedures for participation of local communities in water and sanitation management	SDG Tier III	1. Measurement concept match rating (4/7): This indicator measures the implementation of mechanisms well, but it is specific to water and sanitation management. 2. Technical quality rating (4/8): This continuous indicator is aligned with the SDGs (criteria 5 and 2) which will increase acceptability across countries (criteria 7 and 8). However, given the data in Tier III are not yet available, technical quality is difficult to assess at this point.
	2.2.2 Proportion of cities with a direct participation structure of civil society in urban planning and management that operate regularly and democratically	SDG Tier III	1. Measurement concept match rating (5/7): This indicator measures the implementation of mechanisms in a broader sense that emphasizes participation in policymaking. 2. Technical quality rating (3/8): This regional indicator does not yet have data available (Tier III). However, it is aligned with the SDGs (criteria 2, 7 and 8).
	Indicators align moderately well with the measurement concepts, but have data availability over the long term only.		
2.3 Level of between-country exchange of good practices around participation in policymaking	2.3.1 Whether country has communicated the establishment or operationalization of an integrated policy, strategy, or plan that increases its ability to adapt to the adverse impacts of climate change, and foster climate resilience and low greenhouse gas emissions development in a manner that does not threaten food production (including a national adaptation plan, nationally determined contribution, national communication, biennial update report or other)	SDG Tier III	1. Measurement concept match rating (1/7): This indicator indirectly measures exchange of good practices. It does not address participation in policymaking. 2. Technical quality rating (3/8): This indicator is aligned with the SDGs and thus theoretically has high acceptability across countries. Data are not available.
	2.3.2 Number of least developed countries and small island developing Member States that are receiving specialized support, and amount of support, including finance, technology and capacity-building, for mechanisms for raising capacities for effective climate change-related planning and management, including focusing on women, youth and local and marginalized communities	SDG Tier III	1. Measurement concept match rating (0/7): This indicator does not align with the measurement concept. 2. Technical quality rating (3/8): This indicator is aligned with the SDGs and thus theoretically has high acceptability across countries. Data are not available.
	Neither indicator is a good match with measurement concept nor are data available. Further work is needed to identify other potential indicators.		
	OVERALL ASSESSMENT of DOMAIN 2 The only indicator recommended at this time is 2.1.1 (*Whether country has adopted and implemented constitutional, statutory or policy guarantees for public access to information*) because it aligns best with the measurement concept and has high-quality data available in the short-term. If data cannot be accessed in time, indicator 2.1.3 (*Whether country has met its commitments and obligations in transmitting information as required by each relevant agreement on hazardous waste and other chemicals*) whichever is more specific can be used as a proxy because data are available. For all other measurement concepts, no indicators aligned well with measurement concepts and had high-quality data available.		
Domain 3: Health system reorientation			
3.1 The level of comprehensive, [equitable] service coverage by health systems (including primary health care and the right to health) ALTERNATIVE MEASUREMENT CONCEPT [Inequalities in the level of comprehensive service coverage by health systems]	3.1.1. *[SDG 16.9.1]* Proportion of children under 5 years of age whose births have been registered with a civil authority, by age [Gender disaggregation is possible with this indicator; therefore, a parity index between females and male registration could be used to examine inequalities in comprehensive service coverage by health services	SDG Tier I	1. Measurement concept match rating (4/7): This indicator addresses an important vehicle for demanding the right to health services. Several complications exist, however, that need to be addressed in thinking through the match. These may arise from the formulation of the measurement concept. The first complication is whether equity should be included in the measurement concept or measured separately, making the measurement focus on *comprehensive, implying the full spectrum of care (including addressing key food, water and other environmental determinants as identified as part of primary health care and the right to health).* For this reason, the *measurement concept – indicator* match rating was considered moderate. 2. Technical quality rating (4/8): As Tier I, a suggested methodology exists that has been tested and is an international standard; therefore, it meets criterion 1 (SMART). It is feasible and acceptable given alignment with SDG indicators and the associated process used for international agreement. It would also therefore meet criteria 2,3,7, and 8. Some evidence regarding the usefulness of this indicator-concept in being associated with access to determinants for health equity is available but needs further documentation (criterion 6). The criterion about the indicator being continuous is fulfilled. Regular availability of data would be fulfilled as part of the SDGs (criterion 4).
	3.1.2. *[SDG 6.1.1]* Percentage of population using safely managed drinking water services [Regarding inequalities, as with the previous indicator of 3.1.1. parity indices by rural/urban and by lowest/highest wealth quintiles could be constructed on the basis of available data.]	SDG Tier I	1. Measurement concept match rating (6/7): A single rating process was adopted here because the right to drinking water, sanitation and safety from harmful exposures are included in the right to health and the policies for primary health care. For this reason, the *measurement concept – indicator* match rating was considered moderate to high. 2. Technical quality rating (6/8): As Tier I, a recommended methodology exists that has been tested and is an international standard; therefore, indicators 3.1.2. and 3.2.3. meet criterion 1 (SMART). They are feasible and acceptable given alignment with SDG indicators and the associated process used for international agreement. They would also therefore meet criteria 2,3,7, and 8. Evidence regarding the usefulness of this indicator-concept in being associated with access to determinants for health equity is available but needs further documentation (criterion 6). The criterion about the indicator being continuous is fulfilled given the “coverage” nature of this indicator. Regular availability of data would be fulfilled as part of the SDGs (criterion 4). An overall technical rating of 8/8 was given. Other considerations: a hierarchy exists in the indicator concepts and their alignment with the measurement concept. If so, water may be prioritized, or a combined index could be created from individual level data (percentage of population with coverage in 1,2 and 3). This indicator has been tested previously and would require a limited data collection burden. One advantage would be to increase relevance in countries with high water provision rates because sanitation often lags behind water provision.
	3.1.3. *[SDG 6.2.1]* Percentage of population using safely managed sanitation services including a hand washing facility with soap and water [Regarding inequalities, as with the previous indicator of 3.1.1. parity indices by rural or urban and lowest or highest wealth quintile could be constructed on the basis of available data.]	SDG Tier I	
	3.1.4. *[SDG 6.3.1]* Percentage of wastewater safely treated	SDG Tier III	1. Measurement concept match rating (5/7): A link exists between the responsibility of public and private companies to maintain a safe and healthy environment and the health system actions for prevention and health promotion in light of Alma Ata Declaration and the right to health. In particular, unsafe water inequitably distributed resulted in deteriorated environments for more disadvantaged groups. For this reason, the *measurement concept – indicator* match rating was considered moderate to high. 2. Technical quality: (2/8) Because of technical problems with data collection, this indicator should be excluded for now.
	*The SDH emphasis in universal coverage envisaged by the measurement concept relates to pledge 3.2 of the Rio Political Declaration. [Strengthen health systems towards the provision of equitable universal coverage and promote access to high-quality, promotive, preventive, curative and rehabilitative health services throughout the life-cycle, with particular focus on comprehensive, integrated primary health care.]* A key feature of the pledge is the comprehensive nature of health systems – stretching along the care continuum, including primary health prevention and promotion services as described in the primary health care movement of the Alma Ata Declaration and in the right to health. Another key feature is equity. In view of this combined focus, the best possible combination of indicators to measure this construct would be two parity indices, one related to identify (gender parity) and one related to urban/rural parity for a combined coverage indicator of drinking water and sanitation. The question as to whether these indicators are sufficient to describe the full measurement concept needs some discussion.		
3.2 Level of integration of equity into health systems, policies and programs	3.2.1. Country Policy and Institutional Assessment (CPIA) policies for social inclusion or equity for gender equality, equity of public resource use, building human resources, social protection and labor, and policies and institutions for environmental sustainability (average from a 1 = low to 6 = high) [The Country Policy and Institutional Assessment (CPIA) rates countries against a set of 16 criteria grouped in four clusters: (a) economic management; (b) structural policies; (c) policies for social inclusion and equity; and (d) public sector management and institutions.]	World Bank [see description]	1. Measurement concept match rating (2–4/7): The *measurement concept – indicator* match rating was considered moderate. An assumption exists that the social and public health institutions will move in parallel. Some support might exist for this in institutional theory. However, further study of this indicator would be needed to assess how this indicator covers the private sector, and associated resource use. It needs further assessment based on our criteria and more information on the exact focus and construction. The element of *Equity of Public Resource Use* could be particularly interesting: “Criterion assessing the consistency of government spending with the poverty reduction priorities taking into account the extent to which: (a) individuals, groups, or localities that are poor, vulnerable, or have unequal access to services and opportunities are identified; (b) a national development strategy with explicit interventions to assist the groups identified in (a) has been adopted; and (c) the composition and incidence of public expenditures are tracked systematically and their results fed back into subsequent resource allocation decisions. The assessment of the revenue collection dimension takes into account the incidence of major taxes, e.g., whether they are progressive or regressive, and their alignment with the poverty reduction priorities.” A potential problem with this indicator is the inclusion of multiple sectors and being able to extract the health component. When relevant, expenditure and revenue collection trends at the national and subnational levels should be considered. 2. Technical quality: the indicator covers 95 countries dating back (not for all) to 2005. Further assessment regarding criteria is needed after establishing how the health sector component can be extracted.
3.3 Level of knowledge exchange on equity-oriented policies and programs	None available.		
	OVERALL ASSESSMENT DOMAIN 3 Overall, weakness exists in the extent of indicators presented to cover reorientation of the health sector. In particular, two out of three measurement concepts do not appear to have feasible indicators with good conceptual matches. No indicator has yet been identified for knowledge exchange regarding equity-oriented policies and programs. Consideration may be needed with regard to the latter concept; perhaps the WHO program budget indicator could be used on a temporary basis. It refers to: [*Number of] Country [ies] that have implemented at least two WHO-supported activities to integrate gender, equity and human rights in their health policies and programs*] and covers all WHO Member States. Starting to monitor with so few indicators might be unacceptable, unless they are complemented by other governance indicators that relate to intersectorality to address the SDH equity. Other options are to reconsider basic expenditure indicators (as a percentage of gross national product (public versus private (for-profit). National health accounts definitions need to be consulted. However, some empirical work has linked equity orientation with public health expenditure as a percentage of total health expenditure.		
Domain 4: Global governance			
4.1 Level of implementation of the 1993 *Vienna Declaration and Programme of Action*	4.1.1 Percentage of countries where the legal framework (including customary law) guarantees women’s equal rights to land ownership or control [SDG Indicator 5.a.2]	SDG Tier II (FAO) Tier III (World Bank)	1. Measurement concept match rating (4/7): This indicator is somewhat aligned with the Vienna Declaration and Programme of Action because it measures legislative action on women’s equal rights to land ownership or control. 2. Technical quality rating (5/8): This indicator meets SMART criteria (1) and is aligned with the SDGs (2). This indicator is binary at the country level (yes or no), but continuous at the international level (5). Additionally, this indicator is applicable across countries (7), and is accepted by international organizations such as FAO, the World Bank, and UN Women (8). However, this indicator may be difficult to obtain (3), is not reported annually (4), and may have limited evidence of direct benefit to the SDH. Although FAO has proposed methodology for this indicator, it is not being reported; therefore, data availability is limited.
	4.1.2 Proportion of countries with laws and regulations that guarantee women and adolescents access to sexual and reproductive health services, information and education (official records) [SDG Indicator 5.6.2]	SDG Tier III (UNFPA)	1. Measurement concept match rating (4/7): This indicator is somewhat aligned with the Vienna Declaration and Programme of Action; because it measures legislative action on women’s rights to access sexual and reproductive health services. 2. Technical quality rating (6/8): This indicator meets SMART criteria (1) and is aligned with the SDGs (2). This indicator is binary at the country level (yes/no), but continuous at the international level (5). Access to sexual and reproductive health may have direct benefit to the SDH. Additionally, this indicator is applicable across countries (7), and is accepted by international organizations such as UNFPA (8). However, this indicator may be difficult to obtain (3), and is not reported annually (4), Although UNFPA has proposed methodology for this indicator, it is not being reported; therefore, data are not available.
	Both indicators are a good match with measurement concepts, but have data availability over the long term only.		
4.2 Level of implementation of international agreements that improve the SDH	4.2.1 Number of parties to international multilateral environmental agreements on hazardous waste, and other chemicals who meet their commitments and obligations in transmitting information as required by each relevant agreement [SDG Indicator 12.4.1]	SDG Tier I (UNEP)	1. Measurement concept match rating (6/7): This indicator is directly relevant to the implementation of international agreements that improve SDH, because it measures compliance with environmental agreements on hazardous waste and other chemicals. 2. Technical quality rating (7/8): This indicator meets SMART criteria (1), is aligned with the SDGs (2), is feasible or cost-effective to obtain (3) and can be reported annually (4). This indicator is binary at the country level (yes/no), but continuous at the international level (5). Additionally, this indicator is applicable across countries (7), and is accepted by such international organizations as UNEP (8). However, evidence might be limited that transmitting information about hazardous waste or other chemicals has direct benefit to the SDH (6). UNEP has established methodology for this indicator, and data are currently available.
	4.2.2 Number of countries that have communicated the establishment or operationalization of an integrated policy, strategy, or plan which increases their ability to adapt to the adverse impacts of climate change, and foster climate resilience and low greenhouse gas emissions development in a manner that does not threaten food production (including a national adaptation plan, nationally determined contribution, national communication, biennial update report or other) [SDG Indicator 13.2.1]	SDG Tier III (UNFCCC)	1. Measurement concept match rating (4/7): This indicator is somewhat related to implementation of international agreements that improve SDH, as it measures the establishment and operationalization of an integrated policy, strategy, or plan to adapt to the adverse impacts of climate change. 2. Technical quality rating (5/8): This indicator is aligned with the SDGs (2), is applicable across countries (7) and is accepted by international organization such as the UNFCC (8). This indicator is binary at the country level (yes/no), but continuous at the international level (5). Additionally, establishment and operationalization of an integrated policy, strategy, or plan to adapt to the adverse impacts of climate change may directly benefit the SDH (6). However, this indicator may not be specific or measurable, because the establishment and operationalization of an integrated policy, strategy, or plan to adapt to the adverse impacts of climate change is somewhat subjective. This indicator may be difficult to obtain (3), is not annually reported (4), Although UNFCCC has established methodology for this indicator, it is not currently reported; data are not currently available.
	4.2.3 Number of least developed countries and small island developing Member States that are receiving specialized support, and amount of support, including finance, technology and capacity-building, for mechanisms for raising capacities for effective climate change-related planning and management, including focusing on women, youth and local and marginalized communities [SDG Indicator 13.b.1]	SDG Tier III (WMO)	1. Measurement concept match rating (3/7): This indicator is only tangentially relevant to the implementation of international agreements that improve SDH, because it measures number of least developed countries receiving support for climate change planning and management. 2. Technical quality rating (4/8): This indicator is aligned with the SDGs (2), and accepted by international organizations such as the WMO (8). This indicator is binary at the country level (yes or no), but continuous at the international level; amount of support received is continuous (5). Additionally, support for climate change management may directly benefit the SDH (6). However, this indicator may not be specific or measurable, because support for climate change management (aside from financial support) is somewhat subjective (1). This indicator may be difficult to obtain (3), is not reported annually (4), and is not broadly applicable to all countries (7). No established methodology exists for this indicator, and data are not available.
	All three indicators capture the measurement concept. The candidate indicator 4.2.1. is prioritized, because it has immediate data availability. The prioritized indicator is fit for purpose and does not require further development.		
	OVERALL ASSESSMENT of DOMAIN 4 Only one indicator (4.2.1 Number of parties to international multilateral environmental agreements on hazardous waste, and other chemicals that meet their commitments and obligations in transmitting information as required by each relevant agreement [SDG Indicator 12.4.1]) meets both measurement concept match and technical quality criteria, and can be measured with data that are currently available. Although three of the five remaining indicators meet a substantial amount of measurement concept match and technical quality criteria, data are not available for measuring these indicators. Further work is needed to generate data that will measure the indicators proposed in this domain; additionally, the proposed core indicator does not capture all measurement concepts in this domain.		
Domain 5: Monitoring Progress			
5.1. The level (extent of) of development and analysis of database (s) containing disaggregated data relevant to health determinants and health equity	5.1.1*[SDG 17.18.1]* Proportion of sustainable development indicators produced at the national level with full disaggregation when relevant to the target, in accordance with the Fundamental Principles of Official Statistics	SDG Tier III	Measurement concept match rating (4/7): It covers the idea of disaggregation of data for health determinants and health (goal III). However, it does not refer to the analysis component, which is arguably the equally important aspect. For this reason, the *measurement concept – indicator* match rating was reduced. 2. Technical quality rating (2/8): As Tier III, methodology is recommended but it has not been tested and no international standard exists; therefore, it cannot be assessed for criterion 1 (SMART). It would also therefore not meet criteria 2,3,7, or 8. Evidence is quite good in relation to the availability of disaggregated data to stimulate action on SDH (criterion 6). Continuousness of indicator (criteria 5) should be fulfilled. Regular availability of data would be fulfilled if it is part of the SDGs (criterion 4).
5.2. Promotion and investment in research and evaluations of SDH interventions to promote equity	5.2.1 *[SDG 3.b.2]* Total net official development assistance to the medical research and basic health sectors	Tier I c	1. Measurement concept match rating (3/7): This indicator is being used under Goal III (health) of the SDGs to monitor the target related to “*Support the research and development of vaccines and medicines for the communicable and non-communicable diseases that primarily affect developing countries, provide access to affordable essential medicines and vaccines, in accordance with the Doha Declaration on the TRIPS Agreement and Public Health, which affirms the right of developing countries to use to the full the provisions in the Agreement on Trade-Related Aspects of Intellectual Property Rights regarding flexibilities to protect public health, and, in particular, provide access to medicines for all.”* It covers the idea of official development aid and other resource flows to countries and how they are used for medical research but the breakdown of basic health sectors is still problematic. In supporting the flow of investments from North to South it corresponds to some of the spirit of other Rio pledges related to improving equity between countries. However, the indicator does not measure national allocations to research and evaluations, which would be a much better fitting concept. Also, the Rio pledges it relates to refer to describe not only the production of evaluations but also systematically sharing evidence, as well as using these assessments for guiding policymaking. Because the indicator lacks covering these aspects, the *measurement concept – indicator* match rating was rated low. A further consideration, given its health sector and international focus, is that it may be considered more relevant to reorienting the health sector measurement domain or the global governance domain. 2. Technical quality (6/8) As Tier I, the indicator has a well-established methodology that has been tested and an international standard (criterion 1, SMART). It is feasible and acceptable given alignment with SDG indicators and the associated process used for international agreement (criteria 2,3,7,8). Criterion 5 on continuous indicators is also met. Further work will be needed to assess how well the indicator meets criterion 6 related to impacts on health equity.
	5.2.2 *[SDG 17.16.1]* Country reports progress in multi-stakeholder development effectiveness monitoring frameworks that support the achievement of the sustainable development goals	Tier II	1. Measurement concept match rating (5/7) The notion that multi-stakeholder action (across sectors and across government and civil society) is important in development frameworks is mentioned in the Rio Political Declaration. The indicator (modified to represent the indicator at the country level) tries to measure the effectiveness of these multi-stakeholder processes in relation to achieving SDGs. This could have relevance for health determinants as well although health is not specifically mentioned, given that health is impacted by so many goals, the evaluation of the effectiveness of the framework would need to assess the impacts across different areas of SDGs. On the basis of these assumptions, the *measurement concept – indicator* match rating was rated fairly high. However, more work needs to be done to review the methodology used for describing effectiveness. 2. Technical quality (5/8) As Tier II in SDGs, an established methodology that has been tested and a fair degree of international agreement exists on the methodology (Organization for Economic Co-operation and Development for 80 African countries); therefore, criterion 1 (SMART) roughly meets [although the United Nations Development Programme is proposing another methodology to assess development effectiveness monitoring frameworks that support achievement of the sustainable development goals]. It is feasible given alignment with SDG indicators and the associated process used for international agreement (criteria 2,3, and 7). How acceptable it is might be queried (criterion 8). Further assessment of match to the technical criterion of “preference for continuous indicators (criterion 5)” (e.g., continuous) will need to be completed after obtaining more information on how the indicator is calculated at the national level. As an SDG indicator it would be available routinely (criteria 4). The linkages to health inequities is not yet established (therefore, failing criterion 6).
	Given the deficiencies of the two indicators, selecting between them is difficult. If measurement concept is the overriding consideration, preference must be for the second one SDG 17.16.1] [Number of] countries reporting progress in multi-stakeholder development effectiveness monitoring frameworks that support achievement of the sustainable development goals. However, we recommend that another indicator mighty be sought in relation to use of national research funds.		
5.3. (Level of) access to information as a key component of research, monitoring and evaluationsto ensure accountability and justice	5.3.1. *[SDG 16.10.2]* Country has adopt*ed* and implement*ed* constitutional, statutory, or policy guarantees for public access to information	Tier II	1. Measurement concept match rating: (5/7) It relates to how public access to information produces positive impacts on health determinants; although the rule of law mentioned in *the indicator* provides for populations having access to information, no mention is made of recourse to justice, which is specified in the measurement concept, in cases where information enables them to address determinants affecting health and well-being. For this reason, the *measurement concept–indicator* match rating was reduced. 2. Technical quality rating: (5/8) As Tier II, the indicator has a fairly well-established methodology that has been tested and is an international standard (criterion 1, SMART). It is feasible and acceptable given alignment with SDG indicators and the associated process used for international agreement (criteria 2,3,7, and 8). Evidence is emerging on role of information for SDH, and, in particular, for health service use (criterion 6). Further assessment of match to the technical criterion of “preference for continuous indicators (criterion 5)” (e.g., continuous) will need to be completed after obtaining more information on the SDG proposals for indices, which are being proposed as sourced from either the World Bank Road Infrastructure Development project or from United Nations Educational, Scientific and Cultural Organization. These may be composite indices, in which case the framing of the indicator wording will need to be changed.
	OVERALL ASSESSMENT DOMAIN 5 The only indicator in this domain that meets minimum standards (which need to be developed further but can be thought of at least 5/7 on measurement fit and 5/8) on technical criteria fit, is 5.3.1. *[SDG 16.10.2]* Country has adopt*ed* and implement*ed* constitutional, statutory or policy guarantees for public access to information. Further work needs to be done to assess which other indicators from other data sources can be put forwards for this domain or may need to be developed. Also, it has to be evaluated whether the proposed core indicator is sufficient for an initial core set for monitoring Domain 5.		

*Abbreviations*: *SDG* Sustainable Development Goals, *SDH* social determinants of health, *SMART* specificity, measurability, assignability, realistic, and time-related

## Abbreviations

CSDH: Commission on Social Determinants of Health
PAHO: Pan American Health Organization
PHAC: Public Health Agency of Canada
SDG: Sustainable Development Goals
SDH: Social determinants of health
SMART: Specificity, measurability, assignability, realistic, and time-related
WG: Working Group
WHO: World Health Organization
